# TIMP3 induces gene expression partly through PI3K and their association with vascularization and heart rate

**DOI:** 10.3389/fcvm.2023.1130388

**Published:** 2023-03-28

**Authors:** Zi-Meng Xia, Meng-Yu Song, Yan-Ling Chen, Guozhen Cui, Dong Fan

**Affiliations:** ^1^ Department of Pathology, Zhuhai Campus of Zunyi Medical University, Zhuhai, China; ^2^ Department of Pathophysiology, Zhuhai Campus of Zunyi Medical University, Zhuhai, China; ^3^ Department of Bioengineering, Zhuhai Campus of Zunyi Medical University, Zhuhai, China

**Keywords:** tissue inhibitor of metalloproteinase 3, angiogenesis, heart rate, cardiovascular system, signal pathway, gene expression, phosphoinositide-3-Kinase (PI3K)

## Abstract

**Background:**

Tissue inhibitor of metalloproteinase 3 (TIMP3) was recently demonstrated capable to regulate some gene expression in a myocardial infarction model. Here we aim to explore the gene expression profile in TIMP3-treated cardiomyocytes and related potential cardiovascular functions.

**Methods:**

Total RNA extracted from cultured neonatal rat ventricular myocytes (NRVMs) were used for RNA sequencing analysis and real-time PCR. KEGG pathway enrichment assay and Ingenuity Pathway Analysis (IPA) were performed to study the signaling pathways and downstream effects. Western blot was used to detect phosphorylation of protein kinase B (Akt). A Cell Counting Kit-8 assay was employed to evaluate the proliferation of human umbilical vein endothelial cells (HUVECs). Contraction rate of NRVMs was measured with microscopy.

**Results:**

RNA sequencing data showed that expression of 2,526 genes were significantly modulated by recombinant TIMP3 (rTIMP3, 100 ng/ml) in NRVMs. Some differentially expressed genes (DEGs) were validated with real-time PCR. Several KEGG pathways including the phosphoinositide-3-kinase (PI3K)-Akt pathway were significantly regulated by rTIMP3. Phosphorylation of Akt was increased by rTIMP3 and a PI3K inhibitor LY294002 suppressed rTIMP3-induced up-regulation of some genes. Some DEGs were predicted by IPA to increase vascularization, and some to decrease heart rate. RTIMP3 could reduce the contraction rate of NRVMs and its conditioned media increased the proliferation of HUVECs.

**Conclusion:**

TIMP3 can regulate expression of multiple genes partly through PI3K. Some DEGs were associated with activation of vascularization and some with heart rate reduction. This study suggests that TIMP3 can potentially modulate cardiovascular functions *via* DEGs.

## Introduction

Tissue inhibitor of metalloproteinase 3 (TIMP3) is a member of the TIMP family (TIMP1–4). As a physiological inhibitor of matrix metalloproteinases (MMPs) and a disintegrin and metalloproteinases (ADAMs), TIMP3 can also interact with a variety of molecules or receptors, but the roles of the interactions remain largely unknown (1–5). Deficiency of TIMP3 can worsen cardiac remodeling and dysfunction after myocardial infarction (MI) (6–8), which can be ameliorated by supply of TIMP3 through overexpression or recombinant protein (1, 9–13). Interestingly, there is a dose-dependent effect for TIMP3 on angiogenesis (9). Recombinant TIMP3 (rTIMP3) at and above 1000 ng/ml can competitively inhibit the binding of vascular endothelial growth factor (VEGF) to its receptor 2 (VEGFR2) and suppress VEGF-induced angiogenesis (2, 9), however, rTIMP3 at 10 and 100 ng/ml can enhance VEGF-induced endothelial sprouting with unknown mechanism (9). TIMP3 can also interact with Type-2 angiotensin II receptor (AGTR2), but silencing AGTR2 did not influence the anti-angiogenic effect of TIMP3 (5). Therefore, the function of the interaction and the pro-angiogenic mechanism of TIMP3 require further investigations.

RTIMP3 can suppress MI-induced mRNA levels of MMP9, monocyte chemoattractant protein-1 (MCP-1) and interleukin 8 (IL8) in porcine MI heart (13). Recently, Boutagy et al. found that a full-length rTIMP3 reduced but an N-terminal TIMP3 enhanced cytokines (mainly interleukins and chemokines) expression in the porcine MI heart (14). RTIMP3 (1000 ng/ml) was also demonstrated capable to reduce expression and phosphorylation of epidermal growth factor receptor (EGFR) and to suppress the proliferation of neonatal mouse cardiomyocytes (15). Compared to apolipoprotein E (ApoE)-knockout mice, double knockout of ApoE and TIMP3 significantly decreased expression of apelin (APLN) and enzymes involved in fatty acid oxidation in the heart (16). Therefore, TIMP3 has capability to regulate gene expression in the heart but the influence varies with different length of TIMP3. So far the whole transcriptional profile in cardiomyocytes regulated by TIMP3 alone remains to be explored.

Therefore, we hypothesized that TIMP3 could affect cardiovascular function through regulating different gene expression. We treated neonatal rat ventricular myocytes (NRVMs) with rTIMP3 at 100 ng/ml and screened mRNA expression with RNA sequencing. Differentially expressed genes (DEGs) were analyzed with KEGG pathway enrichment analysis, as well as Ingenuity Pathway Analysis (IPA) for potential upstream regulators and downstream effects. Contraction rate of NRVMs and the proliferation of human umbilical vein endothelial cells (HUVECs) were also measured. We found that more than 2,000 DEGs were significantly modulated by rTIMP3. Phosphoinositide-3-kinase (PI3K) and AGTR2 were involved partly in rTIMP3-induced gene expression. Some DEGs were predicted to increase vascularization, and some to reduce heart rate. RTIMP3 could reduce the contraction rate of NRVMs, and the conditioned media of NRVMs treated with rTIMP3 could enhance the proliferation of HUVECs. This is the first proof that TIMP3 can regulate expression of multiple genes in cardiomyocytes which may mediate its cardiovascular functions.

## Materials and methods

### Isolation and culture of neonatal rat ventricular myocytes

Neonatal rat ventricular myocytes (NRVMs) were isolated and cultured as described previously (17–19) with minor modifications. In brief, ventricles from neonatal (1–3 days old) Sprague-Dawley rats were minced and digested in Hanks’ Balanced Salt Solution (HBSS, Gibco, United States) containing collagenase type II (Worthington, United States) and trypsin (Gibco). After digestion, the cells were pre-incubated in culture media (DMEM with addition of 1% penicillin/streptomycin and 10% FBS) for 90 min to exclude non-myocytes. Unattached myocytes were seeded to new culture dishes or multi-well plates with culture media for 48 h. All myocytes were treated with rTIMP3 (RayBiotech, United States) or phosphate-buffered saline (PBS) after serum-deprivation for 24 h. All experimental procedures involving animals were in accordance with the protocol approved by the Animal Care and Use Committee of Zunyi Medical University.

### Real-time polymerase chain reaction

Total RNA was extracted using TRIzol Reagent (Invitrogen, United States) according to its manufacturer's instructions. And cDNA was produced from the total RNA by using the RevertAid First Strand cDNA Synthesis Kit (Thermo Scientific, United States). Real-time polymerase chain reaction (PCR) was done with the BeyoFast™ SYBR Green qPCR Mix kit (Beyotime, China) using QuantStudio 5 (Applied Biosystems). Relative expression levels of mRNAs were calculated with the 2^−^*^ΔΔ^*^CT^ method (20, 21). The primers used in the real-time PCR were designed with an online Primer-BLAST tool (NCBI) (18, 22–24) and listed in the Supplementary Table S1.

### RNA sequencing, upstream regulators and downstream function analysis

The RNA samples were sent to Novogene (China) for RNA sequencing on an Illumina NovaSeq 6,000 platform. The raw sequence reads were deposited in the NCBI Sequence Read Archive (SRA) database and the accession number is PRJNA923808. Differentially expressed genes (DEGs) were those with |fold changes| ≥2 and *p.adjust *< 0.05. Kyoto Encyclopedia of Genes and Genome (KEGG) pathway enrichment analysis was performed with the DEGs to identify related signaling pathways. Upstream regulators and downstream functions of the DEGs were evaluated by using Upstream Regulator Analysis and Downstream Effects Analysis *via* Ingenuity Pathway Analysis (IPA, version 81348237, Qiagen) software. Potential KEGG pathway enrichment and downstream cardiovascular system development and function were illustrated with an R package ggplot2 (25). Heat maps showing the changes of related DEGs were generated using TBtools software (26).

Gene set enrichment analysis (GSEA) (27, 28) was also employed to determine the enrichment of rat KEGG pathway gene sets with modest but coordinated changes comparing control and rTIMP3 groups. Enrichment score (ES), normalized ES, nominal *p* value and false discovery rate (FDR, *q* value) were calculated by using a GSEA program (v4.3.2) developed by the Broad Institute and a WEB-based Gene SeT AnaLysis Toolkit (WebGestalt) as reported (27–29).

### Western blot

Total protein was extracted from NRVMs with RIPA Lysis Buffer (Beyotime, China). Western blots for phosphorylated protein kinase B (*p*-Akt), total Akt and glyceraldehyde-3-phosphate dehydrogenase (GAPDH) with their corresponding antibodies (Cell Signaling Technology, United States) were performed as previously described (17, 30). The band intensity was measured with ImageJ software (National Institutes of Health, United States) and the ratio of *p*-Akt to total Akt was calculated.

### Cell area measurement of neonatal rat ventricular myocytes

NRVMs were treated with or without rTIMP3 (100 ng/ml). Then images of NRVMs were captured at 24 and 48 h by using microscopy (Olympus IX73) and Olympus cellSens Dimension software. Cell area was measured by ImageJ software.

### Contraction rate of neonatal rat ventricular myocytes

NRVMs were treated with or without rTIMP3 (100 ng/ml) for 48 h and then myocyte contractions were observed and recorded by using microscopy (Olympus IX73) and Olympus cellSens Dimension software. Number of deflections per minute was counted as the rate of contractions as described (31).

### Proliferation of human umbilical vein endothelial cells

Human umbilical vein endothelial cells (HUVECs) were purchased from iCell Bioscience Inc. (China) and cultured in the Endothelial Cell Medium (Sciencell Research Laboratories, United States). After 24 h of serum deprivation, HUVECs were incubated with the conditioned media from the NRVMs treated with or without rTIMP3 for 48 h. After 24 h, proliferation of HUVECs was measured with a Cell Counting Kit-8 (CCK-8, Beyotime, China) assay according to its manufacturer's instructions.

### Statistical analysis

Data were presented as mean ± standard deviation (SD). Student's *t*-test and one-way ANOVA followed by Bonferroni's multiple comparisons test were performed using GraphPad Prism software (V7.0). Statistical significance was considered at *p *< 0.05.

## Results

### The gene expression profile induced by TIMP3 in neonatal rat ventricular myocytes

**Figure 1:**
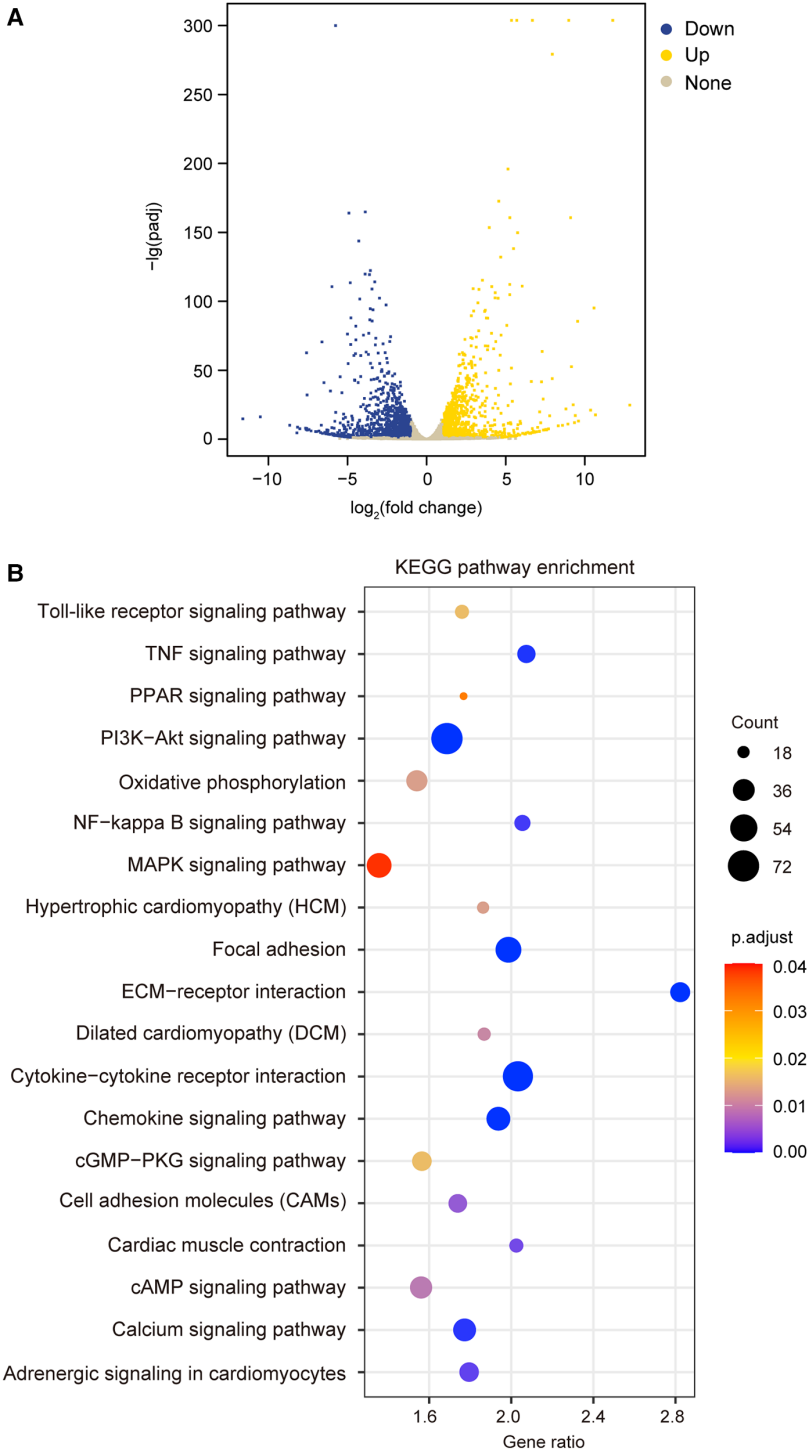
Recombinant TIMP3 (rTIMP3) modulated differential gene expression and potential pathways involved. (**A**) A volcano picture shows differentially expressed genes (DEGs) regulated by rTIMP3, including up-regulated (Up) and down-regulated DEGs (Down) with change fold ≥2 and *p.adjust *< 0.05 and other genes without significance (None). (**B**) KEGG pathway enrichment figure shows some pathways that were significantly regulated by rTIMP3 through DEGs in cardiomyocytes.

**Figure 2:**
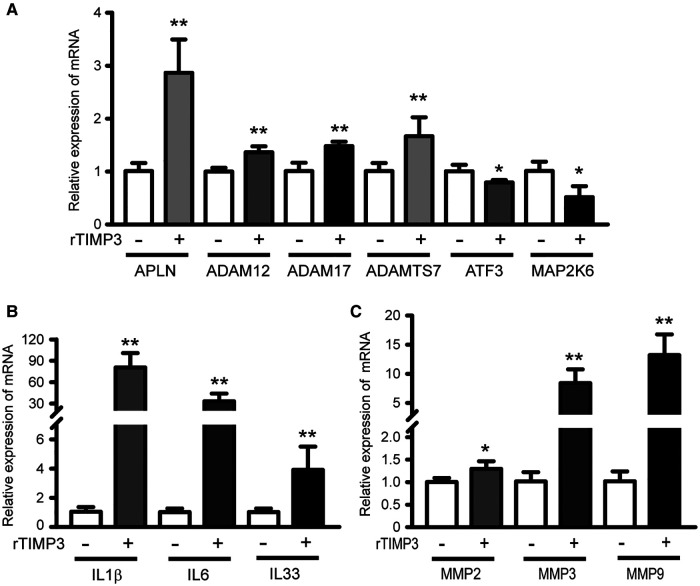
Some DEGs regulated by rTIMP3 were verified by real-time PCR. Neonatal rat ventricular myocytes were treated with rTIMP3 (100 ng/ml) and mRNA levels (*n* = 3), were determined with real-time PCR. ADAM, a disintegrin and metalloproteinase; ADAMTS7, ADAM with thrombospondin motifs 7; APLN, apelin; ATF3, activating transcription factor 3; IL, interleukin; MAP2K6, dual specificity mitogen-activated protein kinase kinase 6; MMP, matrix metalloproteinase. *p < 0.05, **p < 0.01 vs. control.

To test the hypothesis that TIMP3 could regulate different gene expression in cardiomyocytes, we treated isolated NRVMs with or without 100 ng/ml rTIMP3, and then RNA levels were detected by RNA sequencing. Compared with the control group, 2,526 differentially expressed genes (DEGs) with |fold change| ≥2 and *p *< 0.05 were found in the rTIMP3 group. Among the DEGs, 1,062 genes were up-regulated and 1,464 genes down-regulated, which were shown in a volcano illustration (Figure 1A). We randomly chose some DEGs and verified their expression levels with real-time PCR. The results showed that ADAM12, ADAM17, ADAMTS7, APLN, interleukin-1β (IL1β), IL6, IL33, MMP2, MMP3 and MMP9 were significantly up-regulated, while activating transcription factor 3 (ATF3) and dual specificity mitogen-activated protein kinase kinase 6 (MAP2K6) were significantly down-regulated (Figure 2), which were consistent with the RNA sequencing data. Therefore, the RNA sequencing data were reliable.

### KEGG pathways regulated by TIMP3

The DEGs regulated by rTIMP3 were used for KEGG pathway enrichment analysis. 19 pathways were significantly modified, including adrenergic signaling in cardiomyocytes, cAMP signaling pathway, cardiac muscle contraction, cytokine-cytokine receptor interaction, dilated cardiomyopathy (DCM), extracellular matrix (ECM)-receptor interaction, focal adhesion, hypertrophic cardiomyopathy (HCM), mitogen activated protein kinase (MAPK) signaling pathway, nuclear factor-κB (NF-κB) signaling pathway, and PI3K-Akt signaling pathway (Figure 1B). All genes (15,631) with reads higher than zero in RNA sequencing were also analyzed by GSEA with gene sets of rat KEGG pathways, which can detect modest but coordinate alterations comparing two groups (27, 28). Compared to the control group, rTIMP3 significantly up-regulated signaling pathways of chemokine, cytokine-cytokine receptor interaction, MAPK, NF-κB, PI3K-Akt, TNF, and Toll-like receptor, while down-regulated pathways of adrenergic signaling in cardiomyocytes, cardiac muscle contraction, DCM, HCM, and oxidative phosphorylation (Supplementary Table S2).

### Downstream cardiovascular functions of differentially expressed genes regulated by TIMP3

**Figure 3:**
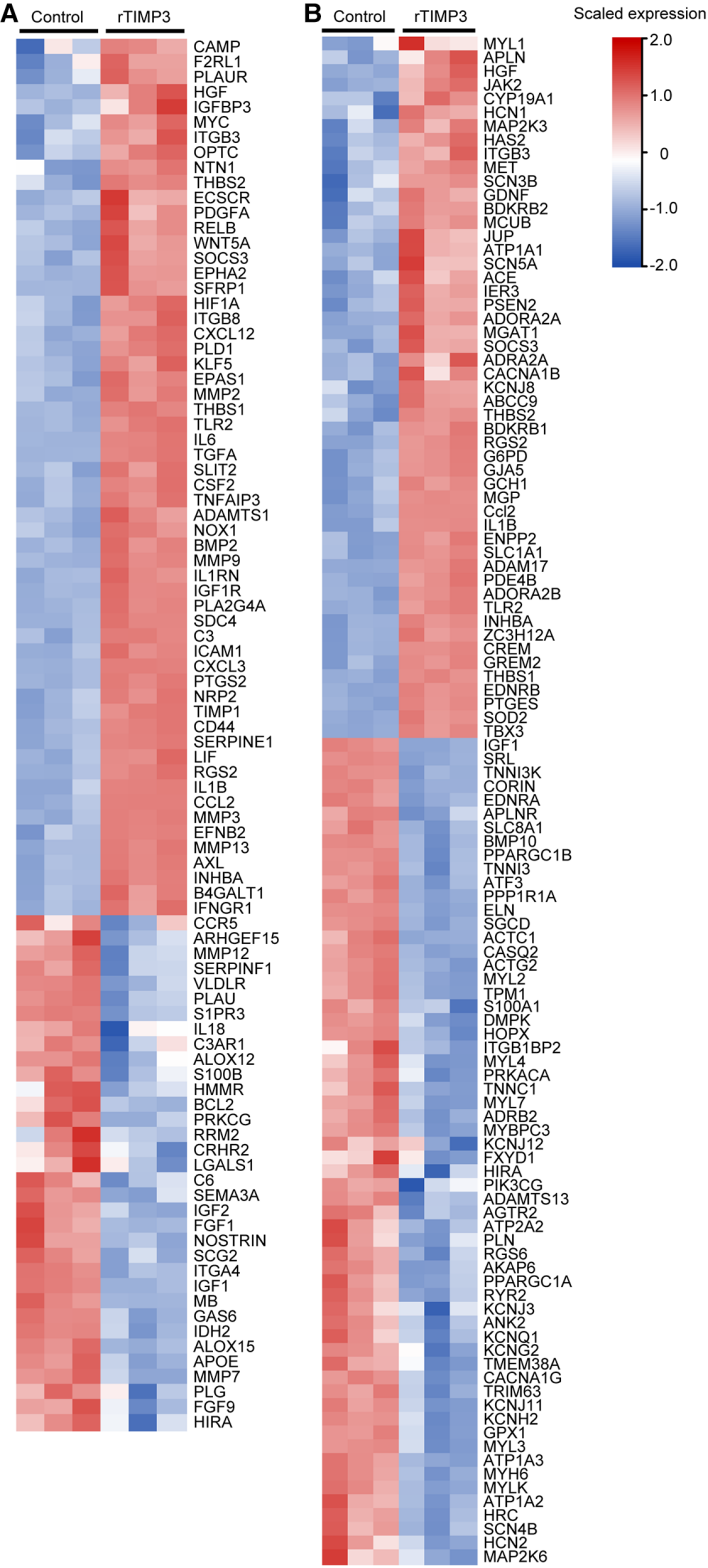
Some DEGs modulated by rTIMP3 were involved in regulation of vascularization and heart rate. DEGs regulated by rTIMP3 were predicted to increase vascularization (**A**) or to decrease heart rate (**B**) *via* Ingenuity Pathway Analysis (IPA) software, and their alterations were presented with heat maps illustrated by using TBtools.

Those DEGs were also used for Downstream Effects Analysis with IPA software. 129 DEGs were predicted to increase cell movement of endothelial cells in cardiovascular system development and function (activation *z*-score = 3.21, *p* < 0.01, Supplementary Figure S1). 92 DEGs including IL1β, IL6, MMP2, MMP3 and MMP9, whose expression levels were verified by real-time PCR (Figure 2), were predicted to increase vascularization (activation *z*-score = 2.04, *p* < 0.01, Supplementary Figure S1), and the DEGs were exhibited in a heat map (Figure 3A). DEGs induced by rTIMP3 also have the potential to positively regulate vasculogenesis (activation *z*-score = 1.90, *p* < 0.01) and angiogenesis (activation *z*-score = 1.60, *p* < 0.01, Supplementary Figure S1). However, rTIMP3 significantly decreased VEGF-D expression and did not significantly affect the expression of VEGF-A, -B, -C and VEGFR2 in NRVMs (data not shown). A recent study showed that paracrine molecules thymosin *β*4 (TMSB4) and prothymosin α (PTMA) released from cardiomyocytes can promote angiogenesis in the infarcted heart (32). In our study, TMSB4 (1.6-fold, *p* < 0.01) but not PTMA was up-regulated by rTIMP3 in cardiomyocytes (data not shown). We also tested the paracrine hypothesis in an *in vitro* experiment. Results showed that the conditioned media from NRVMs treated with rTIMP3 could increase the proliferation of HUVECs compared to control conditioned media (Supplementary Figure S2). This suggests that rTIMP3 may promote angiogenesis/vascularization through paracrine molecules from cardiomyocytes.

Meanwhile, 111 DEGs were predicted to decrease heart rate (activation *z*-score = −2.36, *p* < 0.01) in cardiovascular system development and function (Supplementary Figure S1). The DEGs were illustrated in a heat map (Figure 3B) and some mRNA levels (ADAM17, APLN, ATF3 and MAP2K6) were confirmed with real-time PCR (Figure 2). Then we studied this role with cultured NRVMs. The results demonstrated that rTIMP3 could decrease the contraction rate of NRVMs (Supplementary Figure S3, Supplementary Videos S1–S2). The gene set of cardiac muscle contraction was also significantly down-regulated by rTIMP3 in GSEA analysis (Supplementary Table S2). Therefore, rTIMP3 may reduce heart rate *via* the DEGs found in this project.

As we have found that deficiency of TIMP3 reduced angiotensin II (Ang II)-induced cardiac hypertrophy (33). KEGG enrichment analysis with DEGs regulated by rTIMP3 showed that signaling pathways of HCM and DCM were significantly altered (Figure 1B). GSEA also found that gene sets of HCM and DCM pathways were significantly down-regulated in the rTIMP3 group compared with control (Supplementary Table S2). However, there was no activity pattern available for effects of DEGs on HCM and DCM in the downstream effect analysis *via* IPA software (Supplementary Figure S1). Then we treated NRVMs with rTIMP3 (100 ng/ml) and detected the cell area and mRNA levels of natriuretic peptide A (ANP), BNP, and myosin heavy chain 7 (MYH7). Compared with control, rTIMP3 significantly increased NRVMs area at 24 (Supplementary Figures S4A, B) and 48 h (data not shown), but not the mRNA levels of hypertrophic genes (ANP, BNP and MYH7) at 48 h (Supplementary Figure S4C). It has been reported that physiological cardiac hypertrophy exhibited enlargement of cardiomyocytes with no change or decreased expression of hypertrophic genes (34). Thus, rTIMP3 may trigger physiological hypertrophy in NRVMs.

### Upstream regulators involved in TIMP3-induced gene expression

The DEGs were further used for the Upstream Regulator Analysis in IPA. A variety of molecules or proteins were predicted as upstream regulators, such as IL6, Toll-like receptor 2 (TLR2), NF-κB1, p38 MAPK, ERK1/2, JNK, TLR7, transforming growth factor *α* (TGFA), mothers against decapentaplegic homolog 3 (SMAD3), signal transducer and activator of transcription 3 (STAT3), prostaglandin G/H synthase 2 (PTGS2), PI3K, which were predicted to increase gene expression. Some regulators such as versican core protein (VCAN), B-cell lymphoma 6 protein homolog (BCL6), secreted frizzled-related protein 1 (SFRP1), cbp/p300-interacting transactivator 2 (CITED2), Peroxisome proliferator-activated receptor *γ* coactivator 1-*α* (PPARGC1A), glucocorticoid receptor (NR3C1) were predicted to inhibit gene expression. Some of these regulators/molecules themselves were also regulated by rTIMP3.

**Figure 4:**
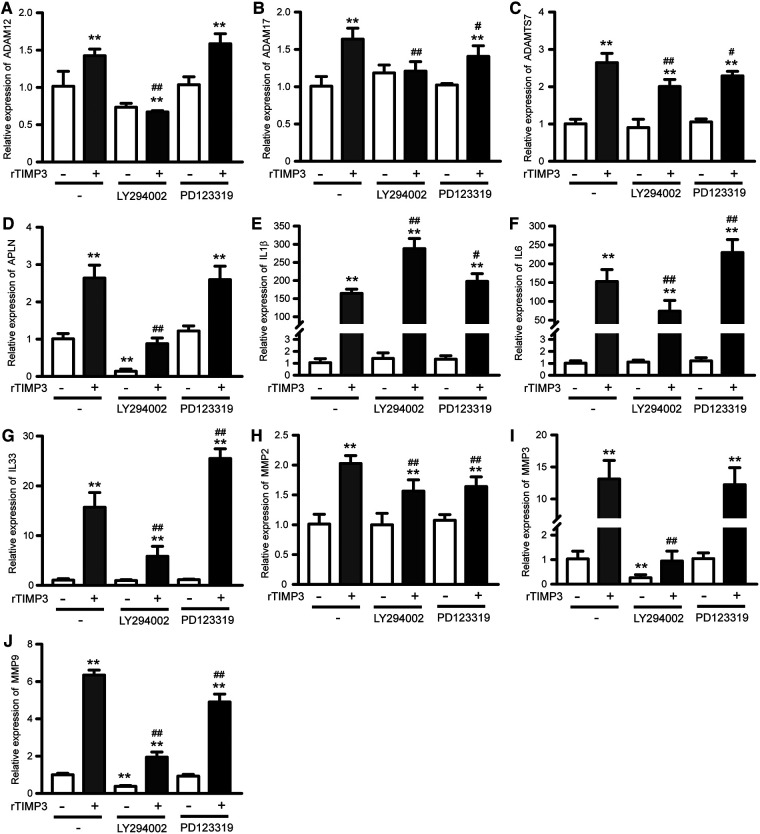
Pi3k and AGTR2 participated in rTIMP3-induced gene expression. Neonatal rat ventricular myocytes were treated with rTIMP3 (100 ng/ml) for 48 h with or without pre-incubation of specific inhibitors for phosphoinositide-3-kinase (PI3K, LY294002, 10 μM) or Type-2 angiotensin II receptor (AGTR2, PD123319, 1 μM) for 30 min. Total RNAs were collected and mRNA levels of ADAM12, ADAM17, ADAMTS7, APLN, IL1β, IL6, IL33, MMP2, MMP3, and MMP9 were detected by using real-time PCR (*n* = 3). **p < 0.01 vs. control, # *p *< 0.05, ## *p *< 0.01, vs. rTIMP3.

As the PI3K-Akt signaling pathway was also found as one of the top KEGG pathways and up-regulated in GSEA analysis, we studied the role of PI3K-Akt in TIMP3-induced gene expression. The phosphorylation of Akt was significantly increased by rTIMP3 in NRVMs (Supplementary Figure S5). And a PI3K inhibitor LY294002 significantly suppressed TIMP3-induced up-regulation of APLN, ADAM12, ADAM17, ADAMTS7, IL6, IL33, MMP2, MMP3 and MMP9, while enhanced TIMP3-induced up-regulation of IL1β (Figure 4). Therefore, PI3K mediated some gene expression induced by rTIMP3. However, the detailed signaling pathway demands further experimental investigations.

AGTR2 has been found to be able to interact with TIMP3 (5). Therefore, we also checked its role in TIMP3-induced gene express by using AGTR2 specific inhibitor PD123319 (P186, Sigma-Aldrich). Results showed that PD123319 did not affect TIMP3-induced up-regulation of APLN, ADAM12 and MMP3. PD123319 suppressed TIMP3-induced up-regulation of ADAM17, ADAMTS7, MMP2 and MMP9, while enhanced the up-regulation of IL1β, IL6 and IL33 (Figure 4). Therefore, the roles of AGTR2 in TIMP3-induced gene expression vary with different genes.

## Discussion

TIMP3 has been found capable to interact with multiple molecules and receptors (1, 3–5), but whether the interactions have impact on gene expression or cardiovascular functions remain largely unknown. So far, most functions of TIMP3 in cardiovascular diseases are mediated primarily by its MMP-inhibitive effect and partly by its anti-inflammatory role (1, 10, 11, 13, 14, 35, 36). Here we demonstrated that rTIMP3 could significantly regulate expression of more than 2,000 genes in cultured NRVMs. Multiple upstream regulators including PI3K were involved in rTIMP3-induced differential gene expression in NRVMs, and rTIMP3 might activate vascularization and reduce heart rate through some DEGs.

TIMP3 has been demonstrated to influence several signal pathways. RTIMP3 (1000 ng/ml) can reduce expression and phosphorylation of epidermal growth factor receptor (EGFR) (15). TIMP3 can competitively bind to VEGFR2 and inhibit its activation (2, 9). TIMP3 can also interact with AGTR2 (5), but the role of the interaction demands further investigation. It has been demonstrated that activation of AGTR2 has negative or no effects on the expression of different genes in the brain tissue (37). Here we found that inhibition of AGTR2 with PD123319 had diverse roles in TIMP3-induced DEGs, including positive (IL1β, IL6 and IL33), negative (ADAM17, ADAMTS7, MMP2 and MMP9), and no effects (ADAM12, APLN and MMP3). Therefore, AGTR2 is partly involved in TIMP3-induced gene expression and its roles depend on specific genes, whereas the detailed mechanisms remain to be further studied.

TIMP3 could inhibit VEGF-induced activation of PI3K in endothelial cells (5) but whether TIMP3 alone can influence the activity of PI3K remain unclear. Recently, Yang et al. found that the PI3K-Akt pathway was promoted by TIMP3 silencing in cervical cancer cells and osteosarcoma cells (38, 39). TIMP3 may suppress the PI3K-Akt pathway *via* up-regulation of phosphatase and tensin homolog (PTEN) (39). Here we demonstrated that the PI3K-Akt pathway was significantly up-regulated by rTIMP3 in GSEA analysis and the phosphorylation of Akt was significantly increased by rTIMP3 in NRVMs. Meanwhile, expression of PTEN was not significantly affected by rTIMP3 in NRVMs (data not shown). Inhibition of PI3K suppressed rTIMP3-induced up-regulation of APLN, ADAM12, ADAM17, ADAMTS7, IL6, IL33, MMP2, MMP3 and MMP9 in NRVMs. Therefore, rTIMP3 can promote expression of some genes through the PI3K-Akt pathway in cardiomyocytes. However, LY294002 further enhanced rTIMP3-induced expression of IL1β. Thus, the role of PI3K in TIMP3-mediated gene expression may vary with different genes and in different cells. IPA also predicted other upstream regulators mediating TIMP3-regulated gene expression, such as MAPK family members, STAT3, TGFA, CITED2, PPARGC1A, and NR3C1, which require further experimental investigations.

KEGG Signaling in HCM and DCM was significantly regulated by rTIMP3, and GSEA revealed that gene sets of both cardiomyopathy were significantly down-regulated, although specific activation pattern could not be found for HCM or DCM with the downstream effect analysis in IPA software. Compared with pathological cardiac hypertrophy, physiological hypertrophy exhibits enlargement of cardiomyocytes with no change or decreased expression of hypertrophic (fetal) genes (34). Here we found that rTIMP3 could induce physiological hypertrophy in NRVMs as the cell area but not the hypertrophic genes was significantly enhanced. It has been demonstrated that exercise-induced physiological cardiac hypertrophy can protect against pathological cardiac hypertrophy and heart failure (40–42). Therefore, it is worthy to study whether rTIMP3-induced physiological cardiomyocyte hypertrophy had such similar cardioprotective effects. However, the response of NRVMs may not be the same as adult cardiomyocytes or the *in vivo* status. Therefore, effects of rTIMP3 on these two types of cardiomyopathy require further experimental studies. Meanwhile, deficiency of TIMP3 has been found capable to suppress Ang II-induced cardiac hypertrophy (33), but to enhance pressure overload-induced DCM primarily due to increased activity of ADAM17, TNF*α* and MMPs (43). Lack of TIMP3 also result in enhanced activation of MMPs and DCM in MI mice (8), which could be suppressed by MMP inhibition or addition of TIMP3 (8, 10, 12, 13). Therefore, the mechanism of TIMP3-modulating cardiac morphology may primarily relate to its MMP inhibitive role.

Angiogenesis or vascularization play a very important role in cardiac repair post MI (9, 44, 45). RTIMP3 at concentration of 1000 ng/ml and above can restrain VEGF-induced angiogenesis through binding to and inhibiting VEGFR2 (2, 9), however, rTIMP3 at 10 and 100 ng/ml can promote VEGF-induced endothelial sprouting with unknown mechanism (9). Overexpression of TIMP3 to some extent can also improve cardiac structure and function post MI by ameliorating adverse cardiac remodeling and promoting angiogenesis (9). These studies indicate that TIMP3 may regulate angiogenesis in a concentration-dependent way. Recent studies showed that paracrine molecules such as VEGF, TMSB4, PTMA, MMP2 and MMP9 released from cardiomyocytes can stimulate angiogenesis during cardiac repair post-MI (32, 46–48). We found that TMSB4 but not PTMA was increased by rTIMP3 in NRVMs. 92 DEGs induced by rTIMP3 (100 ng/ml) in this study were also predicted to promote vascularization and angiogenesis by IPA software. The conditioned media of NRVMs treated with rTIMP3 could increase the proliferation of HUVECs. Therefore, rTIMP3 has the potential to trigger angiogenesis *via* a paracrine way. The DEGs include IL1β, IL6, MMP2, MMP3 and MMP9, but rTIMP3 did not affect the expression of most VEGF family members and even significantly decreased expression of VEGFD. Therefore, TIMP3 may not promote vascularization through VEGF-VEGFR system directly but *via* enhanced expression of inflammatory cytokines and matrix-degrading proteins. On the other hand, TIMP3 can inhibit the enzyme activity of MMPs, which makes the roles of MMPs in vascularization complicated and further investigations are required.

Increased heart rate has been related to poor cardiac function and in-hospital death after MI, and heart rate can be used as a therapeutic target for heart failure (49–51). Recently, reducing heart rate moderately has been proved capable to stimulate cardiomyocyte proliferation under physiological conditions (52). Heart rate reduction can also promote cardiac regenerative repair and angiogenesis after myocardial damage including MI (52). The heart rate was not significantly influenced by deficiency or overexpression of TIMP3 in animal experiments (8, 9), which may be related to that mice were under anesthesia when the heart rate was measured by ultrasound equipment. Therefore, the role of heart rate reduction in TIMP3-mediated protection against cardiac injury remain unclear. It was demonstrated that apelin can reduce heart rate and inducibility of atrial fibrillation (53, 54). Apelin mRNA was reduced in mice with double knockout of ApoE and TIMP3, compared with ApoE-knockout mice (16). And 32-weeks old double knockout mice had more arrhythmic episodes which was detected by using invasive telemetry 24 hour electrocardiography in freely moving mice (16). Therefore, apelin may play an important role in heart rate reduction potentially mediated by TIMP3. In our study, apelin (APLN) was found increased by rTIMP3 and it was included in 111 DEGs that were predicted to reduce heart rate. GSEA also found that the gene set of cardiac muscle contraction was significantly down-regulated by rTIMP3. Meanwhile, the contraction rate of NRVMs was decreased by rTIMP3. Therefore, the 111 DEGs provide potential molecular mechanisms for possible reduction of heart rate by TIMP3.

This study has several limitations or some perspectives to research in future. We used isolated and cultured NRVMs for the gene expression profile, which may be different from the profile in adult cardiomyocytes or the heart *in vivo*. MRNA levels were determined by RNA sequencing and some were confirmed with real-time PCR, but protein levels of the DEGs were to be confirmed. Expression levels of some metalloproteinases, such as MMP-2, −3, −9, ADAM17, were significantly up-regulated, but their activities were not measured. As TIMP3 can also inhibit these metalloproteinases, their precise roles in TIMP3-modulated cardiovascular functions remain to be investigated. We did some *in vitro* experiments to study potential effects of rTIMP3 on vascularization and heart rate, but further *in vivo* experiments are required.

In conclusion, our results demonstrate that rTIMP3 can regulate differential gene expression at least partly through PI3K and AGTR2 in NRVMs. Some DEGs are predicted to activate vascularization and some to decrease heart rate. Meanwhile, rTIMP3 can reduce the contraction rate of NRVMs and its conditioned medium can increase the proliferation of HUVECs. Therefore, our study suggests that TIMP3 can potentially modulate cardiovascular functions through regulation of different gene expression.

## Data Availability

The datasets presented in this study can be found in online repositories. The names of the repository/repositories and accession number(s) can be found in the article/**Supplementary Material**.
