# Supplementary material for: TIMP3 induces gene expression partly through PI3K and their association with vascularization and heart rate
Source: Front Cardiovasc Med. 2023 Mar 28;10:1130388. doi: 10.3389/fcvm.2023.1130388 (PMC10086129; doi:10.3389/fcvm.2023.1130388)
Supplement: Supplementary file 3 [file Datasheet1.pdf]

*Supplementary Material*

# TIMP3 induces gene expression partly through PI3K and their association with vascularization and heart rate

Zi-Meng Xia[1], Meng-Yu Song[1], Yan-Ling Chen[2], Guozhen Cui[3], Dong Fan[1]*

*Correspondence: Dong Fan, dong-fan2010@hotmail.com

Supplementary Table 1. Primers for real-time PCR

| Genes names | Forward primers (5′ → 3′) | Reverse primers (5′ → 3′) |
|---|---|---|
| ADAM12 | GGGATGTGCCTCTTCAACCT | AGCGATTGGTACACTCCTCTG |
| ADAM17 | CACTTTGGTGCCTTTCGTCC | AGAATCAAGCTTCTCAAGTCGC |
| ADAMTS7 | TGGCATTTGCAAGAAAGTGGG | TGAGAATCTCTCGGGCTCCA |
| ANP | AGTGCGGTGTCCAACACAGAT | TTCTCCTCCAGGTGGTCTAGCA |
| APLN | CATGCCTTTCTAAAGCAGGATTG | ACCATCAGCAGCGATAACAGG |
| ATF3 | CCGAGCGAAGACTGGAGCAAA | AGGACATCCGATGGCAAAGG |
| BNP | GACGGGCTGAGGTTGTTTTA | AGAGCTGGGGAAAGAAGAGC |
| GAPDH | TGCACCACCAACTGCTTAG | GATGCAGGGATGATGTTC |
| IL1β | CAGCTTTCGACAGTGAGGAGA | TGTCGAGATGCTGCTGTGAG |
| IL6 | GTTTCTCTCCGCAAGAGACTTC | GGAAGGCAGTGGCTGTCAA |
| IL33 | GACCAGCTATCTCCCATCACT | GGTCTTCTGTTGGGATCTTGTG |
| MAP2K6 | ATGTCTCAGTCGAAAGGCAAG | TCCAAGTCATCGGCCTTCAC |
| MMP2 | CGACGTAACTCCACTACGCT | TCCATCTCCATGCTCCCATC |
| MMP3 | GGAATGGTCTTGGCTCATGC | AGGAATAGGTTGGTACCTGTGAC |
| MMP9 | TCGGATGGTTATCGCTGGTG | TTACAGTGACGTCGGCTCG |
| MYH7 | CACTCCAGAAGAGAAGAACTCCA | TTGTCAGCTTCTTCCGTGCC |

ADAM, a disintegrin and metalloproteinase; ADAMTS7, ADAM with thrombospondin motifs 7; ANP, natriuretic peptide A; APLN, apelin; ATF3, activating transcription factor 3; BNP, natriuretic peptide B; GAPDH, glyceraldehyde-3-phosphate dehydrogenase; IL, interleukin; MAP2K6, dual specificity mitogen-activated protein kinase kinase 6; MMP, matrix metalloproteinase; MYH7, myosin heavy chain 7.

Supplementary Table 2. KEGG pathways enriched from gene set enrichment analysis (GSEA) in neonatal rat ventricular myocytes treated by rTIMP3 (100 ng/ml) compared to control.

| Name | Size | ES | NES | NOM *p*-val | FDR *q*-val |
|---|---|---|---|---|---|
| Toll-like receptor signaling pathway | 78 | 0.583 | 2.258 | <0.001 | <0.001 |
| TNF signaling pathway | 104 | 0.703 | 2.867 | <0.001 | <0.001 |
| PPAR signaling pathway | 59 | -0.365 | -1.260 | 0.124 | 0.271 |
| PI3K-Akt signaling pathway | 285 | 0.333 | 1.524 | <0.001 | 0.041 |
| Oxidative phosphorylation | 97 | -0.691 | -2.645 | <0.001 | <0.001 |
| NF-kappa B signaling pathway | 86 | 0.531 | 2.055 | <0.001 | <0.001 |
| MAPK signaling pathway | 258 | 0.380 | 1.732 | <0.001 | 0.010 |
| Hypertrophic cardiomyopathy | 78 | -0.448 | -1.634 | 0.002 | 0.033 |
| Focal adhesion | 184 | 0.264 | 1.152 | 0.143 | 0.285 |
| ECM-receptor interaction | 74 | -0.333 | -1.216 | 0.143 | 0.317 |
| Dilated cardiomyopathy | 82 | -0.479 | -1.777 | <0.001 | 0.009 |
| Cytokine-cytokine receptor interaction | 168 | 0.622 | 2.699 | <0.001 | <0.001 |
| Chemokine signaling pathway | 150 | 0.562 | 2.399 | <0.001 | <0.001 |
| CGMP-PKG signaling pathway | 139 | -0.316 | -1.263 | 0.063 | 0.270 |
| Cell adhesion molecules | 108 | 0.311 | 1.282 | 0.056 | 0.156 |
| Cardiac muscle contraction | 70 | -0.680 | -2.427 | <0.001 | <0.001 |
| CAMP signaling pathway | 155 | -0.281 | -1.140 | 0.184 | 0.398 |
| Calcium signaling pathway | 183 | -0.356 | -1.472 | 0.009 | 0.090 |
| Adrenergic signaling in cardiomyocytes | 127 | -0.472 | -1.858 | <0.001 | 0.003 |

ES, enrichment score; NES, normalized ES; NOM *p*-val, norminal *p* value; FDR, *q*-val, false discovery rate *q* value.

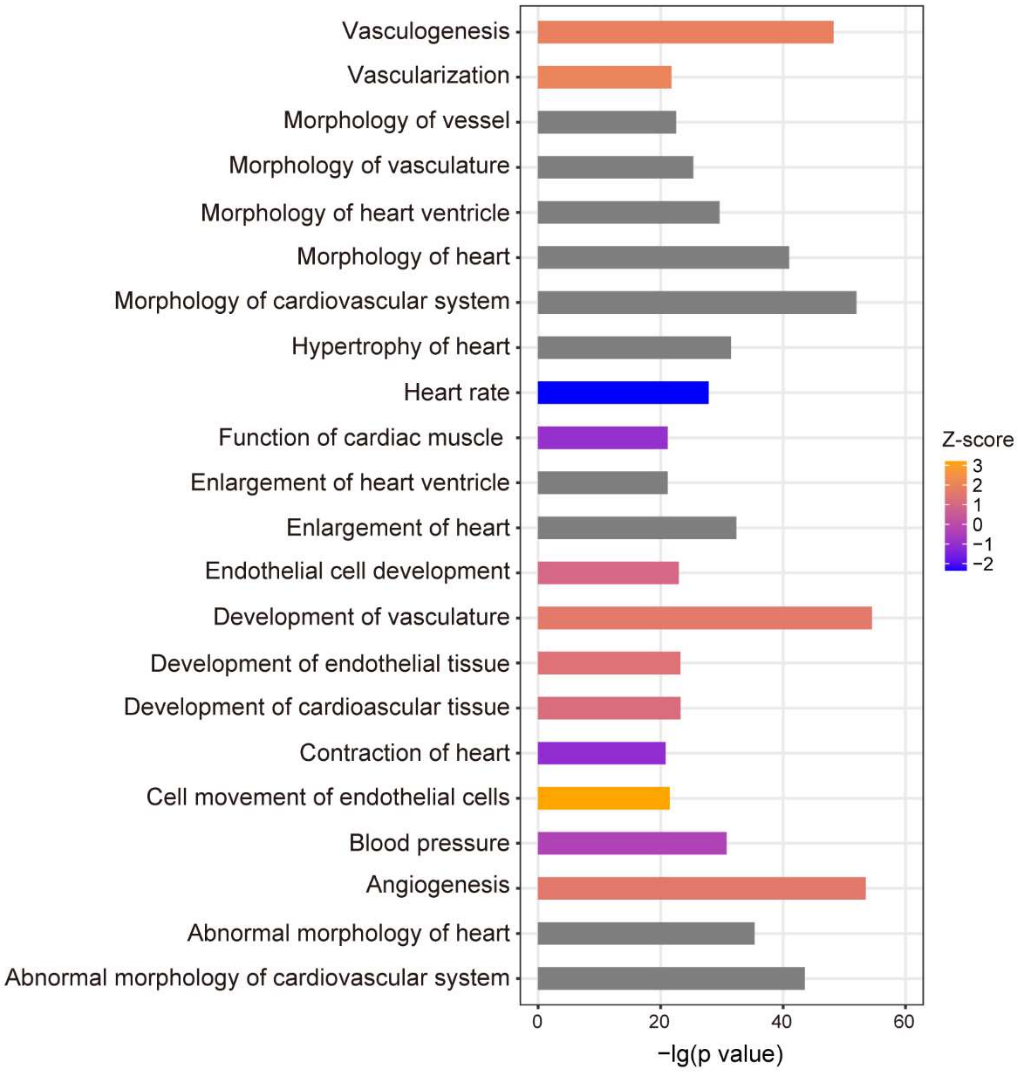


Supplementary Figure 1. The differentially expressed genes (DEGs) modulated by rTIMP3 in cardiomyocytes were predicted to increase cell movement of endothelial cells and vascularization, but to decrease heart rate. Downstream cardiovascular system development and functions related to the DEGs were summarized in the figure. The color in each rectangle indicates its predicted state: increased (orange, positive activation Z-scores), or decreased (blue, negative activation Z-scores), no activity pattern available (grey). The size of the rectangles is correlated with increasing overlap significance [-lg(p value)].

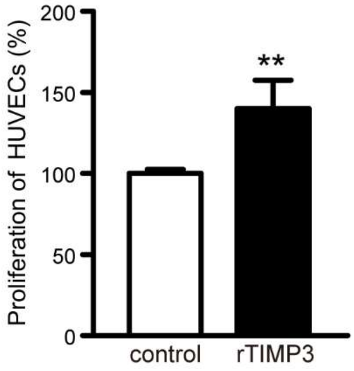


Supplementary Figure 2. Conditioned media from NRVMs treated with rTIMP3 increased the proliferation of HUVECs (mean+SD, n=4). After serum deprivation, human umbilical vein endothelial cells (HUVECs) were cultured in the conditioned media from NRVMs treated with or without rTIMP3. 24 hours later, the proliferation (viability) of HUVECs was detected by a Cell Counting Kit-8 assay. ** $p$<0.01 vs. control.

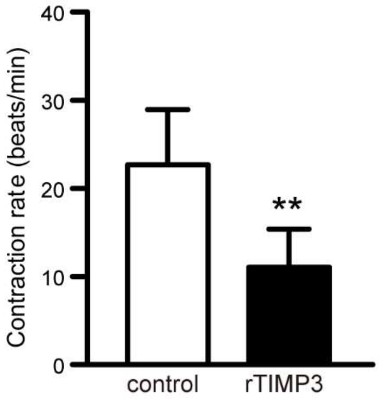


Supplementary Figure 3. The contraction rate of NRVMs was decreased by rTIMP3 (100 ng/ml). NRVMs were treated with or without rTIMP3 (100 ng/ml) for 48 hours. Then contraction of NRVMs was recorded by microscopy and the contraction rate was counted as beats per minute (mean+SD, n=105). ** $p$<0.01 vs. control.

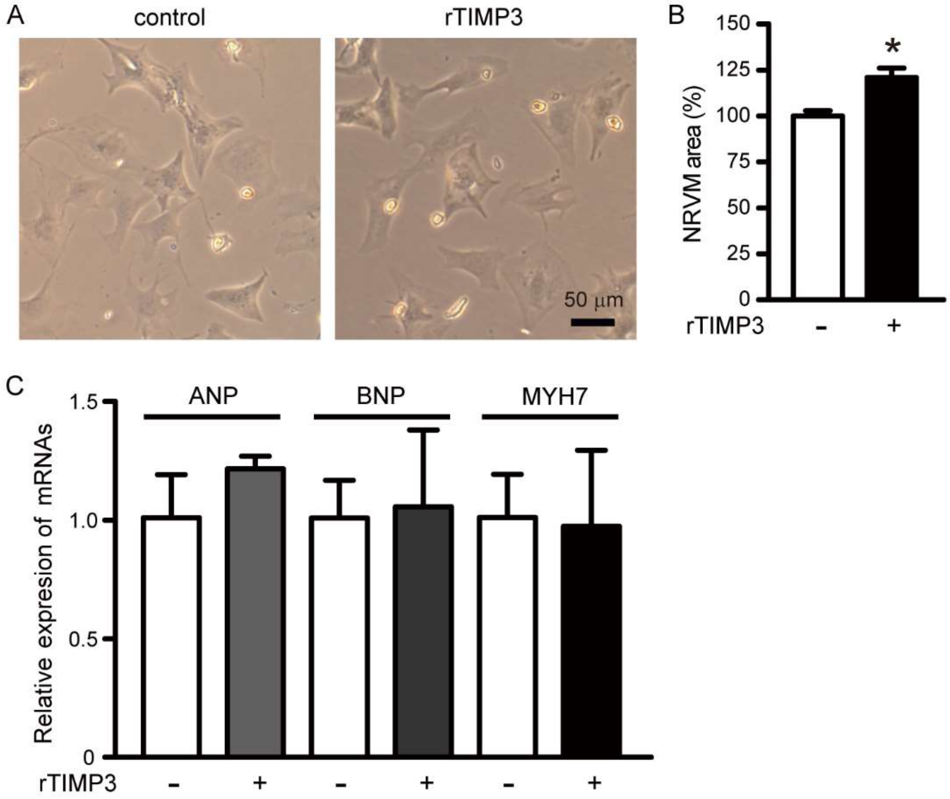


Supplementary Figure 4. RTIMP3 increased NRVMs area but not the mRNA levels of hypertrophic markers. A) Representative images of NRVMs treated with or without rTIMP3 (100 ng/ml), bar=50 μm. B) Statistical data for cell area (normalized to the control group), mean+SEM, n=100 cells/group. C) mRNA levels of natriuretic peptide A (ANP), natriuretic peptide B (BNP), and myosin heavy chain 7 (MYH7) were detected by real-time PCR, mean+SD, n=3. * $p$<0.05 vs. control.

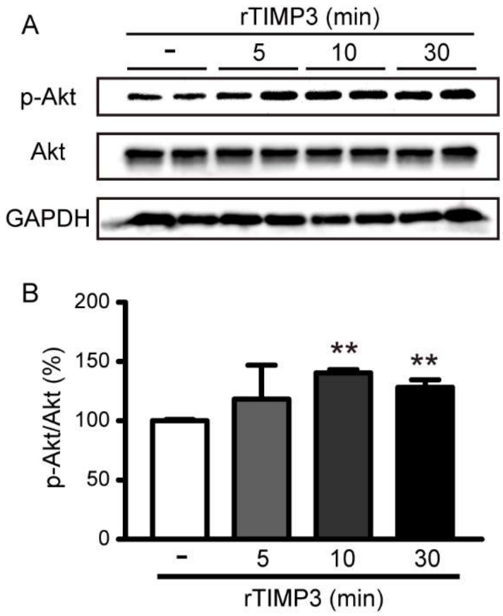


Supplementary Figure 5. The phosphorylation of Akt (p-Akt) was increased by rTIMP3 (100 ng/ml) in NRVMs. A) Representative images of western blots. B) The ratio of p-Akt to total Akt was normalized to the control group (mean+SD, n=3). ** $p$<0.01 vs. control.

Supplementary Video 1. Representative recording of contraction of NRVMs in the control group.

Supplementary Video 2. Representative recording of contraction of NRVMs in the rTIMP3 group.
